# Better estimation of protein-DNA interaction parameters improve prediction of functional sites

**DOI:** 10.1186/1472-6750-8-94

**Published:** 2008-12-23

**Authors:** Vijayalakshmi H Nagaraj, Ruadhan A O'Flanagan, Anirvan M Sengupta

**Affiliations:** 1 BioMaPS Institute, Rutgers University, Piscataway, NJ 08854-8020, USA; 2 The Salk Institute for Biological Studies, La Jolla, CA-92037, USA; 3 Department of Physics and Astronomy, Rutgers University, Piscataway, NJ 08854-8020, USA

## Abstract

**Background:**

Characterizing transcription factor binding motifs is a common bioinformatics task. For transcription factors with variable binding sites, we need to get many suboptimal binding sites in our training dataset to get accurate estimates of free energy penalties for deviating from the consensus DNA sequence. One procedure to do that involves a modified SELEX (Systematic Evolution of Ligands by Exponential Enrichment) method designed to produce many such sequences.

**Results:**

We analyzed low stringency SELEX data for *E. coli *Catabolic Activator Protein (CAP), and we show here that appropriate quantitative analysis improves our ability to predict *in vitro *affinity. To obtain large number of sequences required for this analysis we used a SELEX SAGE protocol developed by Roulet *et al*. The sequences obtained from here were subjected to bioinformatic analysis. The resulting bioinformatic model characterizes the sequence specificity of the protein more accurately than those sequence specificities predicted from previous analysis just by using a few known binding sites available in the literature. The consequences of this increase in accuracy for prediction of in vivo binding sites (and especially functional ones) in the *E. coli *genome are also discussed. We measured the dissociation constants of several putative CAP binding sites by EMSA (Electrophoretic Mobility Shift Assay) and compared the affinities to the bioinformatics scores provided by methods like the weight matrix method and QPMEME (Quadratic Programming Method of Energy Matrix Estimation) trained on known binding sites as well as on the new sites from SELEX SAGE data. We also checked predicted genome sites for conservation in the related species *S. typhimurium*. We found that bioinformatics scores based on SELEX SAGE data does better in terms of prediction of physical binding energies as well as in detecting functional sites.

**Conclusion:**

We think that training binding site detection algorithms on datasets from binding assays lead to better prediction. The improvements in accuracy came from the unbiased nature of the SELEX dataset rather than from the number of sites available. We believe that with progress in short-read sequencing technology, one could use SELEX methods to characterize binding affinities of many low specificity transcription factors.

## Background

Understanding regulatory circuits controlling gene expression is one of the fundamental problems in modern biology. Gene expression is controlled at many different levels but control of transcription is one of the main steps of regulation. One of the best understood control mechanisms is the binding of transcription factors (TFs) to the regulatory sites on DNA in a sequence-specific manner, which affects transcription initiation [1]. The important problem of locating the binding sites for specific TFs, and thus identifying the genes they regulate, has attracted much attention from the bioinformatics community [2,3]. Different methods have been employed for abstracting patterns or "motifs" from the sequences that bind particular TFs leading to predictions of likely binding sites in the genome of the organism under study. Factors regulating multiple genes often have binding motifs low in information content [4], making the task of prediction harder. Examples of such highly pleiotropic proteins range from global regulators in prokaryotes (e. g. CAP, LRP, FIS, IHF, H-NS, HU, *σ *factors [5] in *E. coli*) to Hox proteins [6], important in metazoan development.

Experimental approaches to locating binding sites on DNA [7,8], have uncovered numerous binding sites for various factors. However, looking at the databases devoted to such regulatory sites, like DPInteract [9] and RegulonDB [10] for *E. coli*, SCPD for yeast [11] and TRANSFAC for many higher eukaryotic organisms [12], it is obvious that, for most pleiotropic TFs targeting a large number (100–1000) of genes, the number of known sites is still a small fraction of all the functional sites. A high-throughput version of the chromatin immunoprecipitation method, commonly known as the "ChIP on chip", has been introduced recently [13-15]. In principle, this method locates binding sites genome-wide. However, the resolution is limited to several hundred bases and requires further bioinformatic analysis [16,17].

An alternative approach would be to find the DNA binding specificity of a TF by an *in vitro *method and then use the binding motif to search the genome for putative sites. One of these methods is SELEX [18], which is often used to find the strongest binding sites (sequences close to the consensus) from a library consisting of randomly generated oligonucleotides. However, a TF can often function at binding sites that are far weaker than the consensus. Therefore, to characterize the binding preferences of a TF, we need to identify many of these potential weak binding sites and to estimate the parameters describing the statistical distribution of those sequences. The appropriate modification of the SELEX procedure needed to achieve this goal is based on the SELEX-SAGE procedure [19]. Analysis of the conditions under which we get a significant number of intermediate strength sites was performed in [20]. We will use this procedure on the pleiotropic *E. coli *factor CAP. An alternative to this technology would have been to use DNA chips for protein binding [21,22]. Currently, for transcription factors with long binding sites (e.g. CAP site which is roughly 22 nt), it is common practice to use genomic sequences rather than random libraries in DNA chips. This has its advantages but also might lead to uncertainties regarding the genomic background model in the final statistical analysis.

To abstract a motif from the sequences found by the modified SELEX process, we need a computational method: a supervised algorithm, trained on a set of binding sites identified directly by experimental measurements [23,24,9]. We will compare different supervised methods for extraction of parameters and use CAP targets as a benchmark.

The widely used bioinformatic tool for quantitatively describing such motifs is the weight matrix method [25-29]. Setting the threshold correctly is essential for the quality of predictions (see [9] for an example of strong threshold dependence). However, optimization of the threshold is a non-trivial problem, resolving which is one of the goals of this study. We have shown [4,30] that using the physically correct expression for binding probability, with saturation effects built in, leads to a more accurate estimate for the binding energy and provides a practically useful solution to the problem of classifier threshold choice. The resulting method, Quadratic Programming Method of Energy Matrix Estimation or QPMEME [30], turns out to be a one-class support vector machine [31].

In this paper we do the following:

(i) Perform high throughput, low stringency SELEX experiments.

(ii) Analyze SELEX experiments and extract parameters for models of sequence-dependent TF/DNA interactions by using QPMEME and its extensions.

(iii) Verify *in vitro *affinity predicted by models built on SELEX data by electrophoretic mobility shift assay.

(v) Identify CAP targets in *E. coli*.

(vi) Compare various predicted sites from different methods to see their functional conservation among gram-negative facultative anaerobes.

## Results and discussion

### SAGE SELEX study of CAP

We followed the SELEX-SAGE procedure [19]. After extracting a large number of sequences, we passed these sequences through a quality filter to find sequences that were likely to have been bound by a CAP dimer in the random region of the SELEX pool. We were left with 69 sequences at the end.

### Analysis of sequences from SAGE SELEX study

The maximum-likelihood method for distributions with sharp cutoffs was described in [30]. In particular circumstances, the parameter estimation method becomes a support vector machine, and the resulting algorithm, QPMEME, can be used to determine the binding energy of the protein to the sequence *S*, as *E*(*S*) = ∑_*iα *_*ε*_*iα *_*S*_*iα *_. The variable *S*_*iα *_is defined as follows: if the *i*^th ^base is *α *in sequence *S*, then *S*_*iα *_= 1 and *S*_*iα *_= 0 otherwise. The parameter *ε*_*iα *_is the contribution to the binding free energy from base *α *being at position *i*. These *ε*_*iα *_parameters are chosen to minimize the variance of *E(S) *over the background distribution of sequences *S*, subject to the constraints *E*(*S*^(*j*)^) ≤ -1 for the set of example binding sequences *S*^(*j*)^, *j *= 1, ..., *N*. Sequences satisfying *E*(*S*^(*j*)^) ≤ -1 are then declared to be binding sites. In practice, the base frequencies are taken to be independent and the probability of finding the base *α *is taken to be *p*_*α*_. The quantity to be minimized is given by $\sum_{i\alpha }P_{\alpha }\varepsilon_{i\alpha }^{2}$, subject to the constraints $\sum_{\alpha }P_{\alpha }\varepsilon_{i\alpha }=0$, for each *i*.

The QPMEME algorithm was used to produce an energy matrix, [*ε*_*iα*_], using the set of 49 known CAP binding sites from the DPinteract database. An energy matrix was also constructed using the binding sites identified by the SELEX procedure described below. Weight matrices were also constructed for both the known sites and the SELEX sites using the formula *w*_*iα *_= log [*f*_*iα*_/(*Np*_α_)], where *w*_*iα *_is the *i*, *α *component of the weight matrix and $f_{i\alpha }=\sum_{j}S_{i\alpha }^{\left(j\right)}$ is the frequency of the *i*^th ^base being *α*. The background probabilities for G and C are taken to be the same. The same applies to the background probabilities for A and T. For both algorithms, the background GC content was taken to be 0.43 (the GC content of non-ORF regions in *E. coli*) when constructing energy matrices from the known sites, and was taken to be 0.5 when constructing energy matrices from SELEX sites. For the weight matrices/energy matrices used in the study, see Additional file 1, Additional file 2, Additional file 3 and Additional file 4.

### Prediction of *in vitro *affinity of binding sites

**Figure 1:**
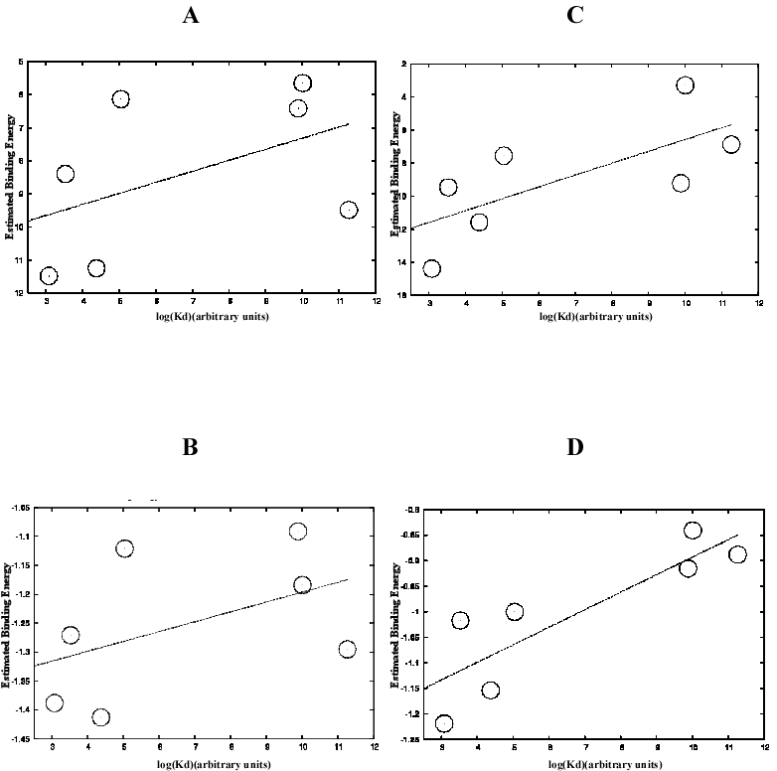
**Estimated binding energy versus log(Kd) with different training sets and methods**. (A) Binding energies inferred using weight matrix method applied to known sites from literature. (B) Binding energies inferred using QPMEME method applied to known sites in literature. (C) Binding energies inferred using weight matrix method applied to SELEX sites obtained by this study. (D) Binding energies inferred using QPMEME method applied to SELEX sites obtained by this study.

The energy matrices constructed using each of the methods allows one to assign an estimated binding energy to a given site. The correlation of these estimated binding energies with the values of log(*K*_*d*_), *K*_*d *_being the dissociation constant, measured for the seven sites (TBS1–6 and the Lac site) as described in the materials and methods section is shown in Fig. 1. From the summary of the correlations in Table 1, it is apparent that the SELEX procedure produced significantly better information about the binding characteristics of CAP than were available using the known sites, while using QPMEME to infer the binding parameters *ε*_*iα *_produced better correlation with the measured values of log(*K*_*d*_) when used with the SELEX data set. The p-values associated with the correlation coefficients (namely the probabilities of getting a correlation coefficient that is greater than or equal to that value for random i. i. d. gaussian data) in table 1 are as follows: r = 0.48 => p = 13.8%, r = 0.71 => p = 3.7%, r = 0.86 => p = 0.65%. The best correlation coefficient, obtained for QPMEME trained on SELEX data, r = 0.86, represents very significant but less than perfect correlation. However, one has to remember that the measurements of *K*_*d*_'s themselves have a certain amount of error. Therefore, even with perfect predictive power, we would not get a correlation coefficient of one.

**Table 1:** Correlation coefficient of inferred binding energy with log(*K*_*d*_)

	Known Sites	SELEX
Weight matrix	0.48	0.71
QPMEME	0.48	0.86

Note that the number of sequences used from the SELEX data is comparable to the number of biological CAP binding sites used in weight matrix determination. This observation suggests that the improvement is due to unbiased sampling of binding sequences and not due to the greater number of sites used.

### Comparison of binding energies of orthologous sites for *E. coli *and *S. typhimurium*

Without evolutionary pressure to keep the binding energy constant over time, the binding energy of the orthologous site will drift towards the average binding energy, which is set to zero in our convention. For an *E. coli *site which is estimated to be a strong binding site, the vast majority of mutations will result in a weaker estimated binding energy. This has the consequence that, even if the true binding energy is conserved, a poor method of estimating binding energies will probably assign a weaker binding energy to the *S. typhimurium *orthologs of those *E. coli *sites with the strongest estimated binding energy.

For each site, *S*, in *E. coli *with an *S. typhimurium *ortholog, *S'*, and for each estimation of the energy matrix, *ε*, one can define the drift of S according to *ε *as:

$$D_{S}=\Theta (E_{2}-E_{1})\frac{E_{2}-E_{1}}{\sigma_{E}}$$

Where *E*_1 _= *ε *• *S*, *E*_2 _= *ε *• *S' *and $\sigma_{E_{1}}$ is the standard deviation of the conditional distribution *P *(*E*_2_|*E*_1_). The function Θ (*x*) is the Heaviside theta function which 1 for *x *≥ 0 and 0 for *x *< 0. The quantity *D*_*s *_measures how much the binding energy of the *S. typhimurium *site has apparently weakened compared to a measure of how much it would be expected to drift if there were no evolutionary pressure to sustain it.

For each of the four estimations of the energy parameters, the total drift for the highest-scoring 200 *E. coli *binding sites with orthologs in *S. typhimurium *is shown in table 2. False positives, that is, candidate binding sites identified in *E. coli*, on the basis of inferred binding energy, which are not functional *in vivo*, would not be expected to have orthologs in *S. typhimurium *with significant binding energy. The overall drift indicated in table 2 then receives contributions from both the false positives picked up during the scan of the *E. coli *genome, and from functional sites whose estimated binding energy differs from the actual binding energy.

**Table 2:** Total drift of 200 strongest *E. coli *binding sites for different methods

	Known Sites	SELEX
Weight Matrix	167	134
QPMEME	139	123

**Figure 2:**
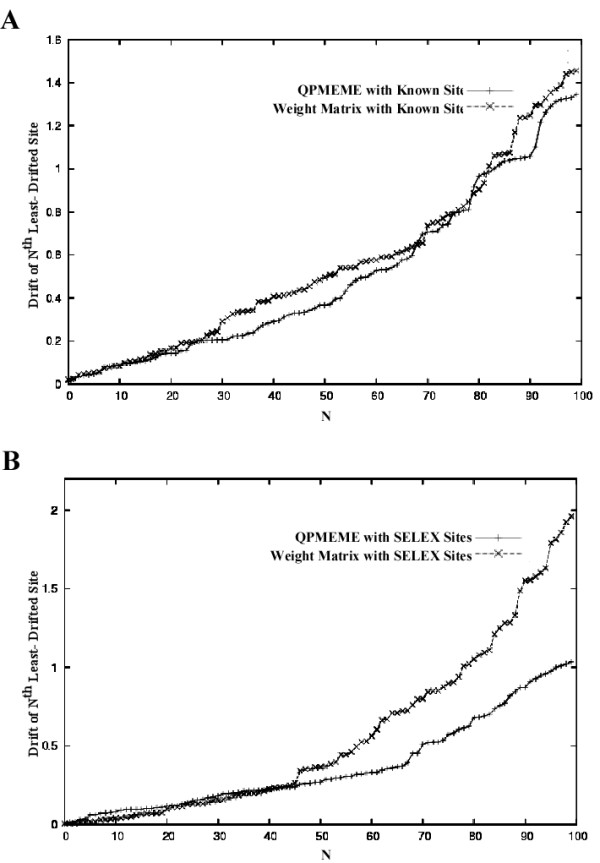
**Drift in estimated CAP binding energy between *E. coli *and *S. typhimurium *sorted in ascending order plotted against the rank**. (A) Using energies estimated by weight matrix or QPMEME based on know sites. (B) Using energies estimated by weight matrix or QPMEME based on the SELEX sites. Note that, for QPMEME estimates based on SELEX data, the energy drift stays low for many sites, as would be expected of most functional CAP targets.

Figures 2a and 2b show the amounts of drift of individual candidate sites ordered according to drift for the energy parameters inferred from the SELEX training set and the training set consisting of the known sites, respectively. Overall, the binding parameters inferred using QPMEME systematically indicated less drift than the parameters inferred using the weight matrix, with the best overall performance when the SELEX data set was used to train the algorithm.

## Conclusion

Our purpose in this analysis was to show that indeed with the appropriate kind of training data, one could improve the ability to predict physical and functional binding. This is in marked contrast to the general feeling in the biology community that many pleoitropic transcription factors bind at too many places and that it is hopeless to try to get functional sites out of motif searches. Recently there were ChIP-chip experiments done on CAP [32]. The conclusion of the study was that CAP is physically bound at many thousands of places in the genome. We saw, however, from the comparative study, that stronger binding sites are significantly conserved, indicating selective pressure. These results are in agreement with similar studies done in yeast [33] based on DNA chip data.

One of the main goals of the experimental procedure was to gather specificity data for a transcription factor (TF) at concentrations comparable to the cellular abundances. One disadvantage of doing the selection experiment with very high abundance of TF is that it is possible to select sequences where the likely binding site partially overlaps with the primer. For low abundances the sites bound tend to be in the variable (meaning, the N28) region, because binding partially to the primer in any window requires enough free energy to be prohibitive. For high abundance, the threshold for tolerance of such energetic costs is higher. We believe that, in the original SELEX SAGE work [19], the TF abundance is still low enough so that the "primer contamination" problem is avoided. However, when using this method for a genuinely pleiotropic TF at cellular concentrations, one would face the primer contamination problem we describe here.

One possible resolution of this problem is computational. We could develop a more complex probability model allowing for the primer contribution and utilize the full data set. We could do this by generalizing the model in reference [30], and allowing binding in different windows on a longer sequence. However, to settle the question of whether SELEX data sets provide any advantage, we decided to focus on small number of sequences where the binding is likely to be in the random or N28 part. The number of SELEX sequences used for training is comparable to the number of biologically known sites. Hence, the improvement in prediction should be from better sampling of sequences in the dataset rather than from the sample size. The use of the full data set requires a new computational method, which would be a promising subject for future research.

In this study, we only used sequence data for estimating the parameters related to the motif. We measured relative affinities (inverse of dissociation constants) to test the accuracy of our predictions. As has been shown, combining SELEX with quantitative affinity measurements leads to even better predictive power [34]. Our reason for focusing solely on sequence data is that we foresee developments in short-read sequencing [35] which is expected to lead to a readily available inexpensive technology for generating large SELEX data sets.

In the last few years, we have seen considerable activity that centers around biophysical aspects of gene regulation. The push has come from two different ends. On the one hand, detailed structural modeling of protein-DNA interactions has been used to calculate sequence dependent protein DNA interaction free energies [36]. The insights from structural considerations could guide the appropriate parametrization of knowledge-based bioinformatic motif discovery tools as well [37]. On the other hand, Hidden Markov Models, which can be thought of as one dimensional statistical mechanics models of multiple proteins binding on DNA, have been applied to study multiple binding sites for protein complexes in a stretch of DNA [38,39]. This approach has been extended to nucleosome positioning as well [40]. The remarkable success of these approaches seems to suggest that much could be done with an accurate biophysical description of protein-DNA interaction in the context of gene regulation. Precise characterization of the probabilistic protein DNA interaction code is a crucial element of such a description. As technologies for massively parallel signature sequencing [35] become more accurate, large scale SELEX studies for determining the interaction code would become more and more feasible.

## Methods

### Purification of CAP

His6-tagged CAP protein was expressed using BL21DE3 cells harboring pAKCRP-HIS6 [41] and purified under native conditions using Ni-NTA Agarose, with slight modifications. Specifically, the elutions were performed with an Imidazole step gradient, with steps of of 60, 100, 200 and 400 mM Imidazole [42]. The pure fractions were dialyzed against a buffer containing 20 mM Tris.cl pH 8.0, 0.1 mM EDTA, 50 mM Nacl, 10% glycerol, 1 mM DTT and 0.1 mM PMSF.

### High throughput low stringency SELEX for CAP

*In vitro *selection, amplification and cloning of TF-binding sites for CAP were implemented using a modified SELEX procedure [18]. 25 nM CAP was used to select binding sequences from a random DNA library N(28) flanked by the PCR primer sequences 5'-CTGTATGTCGAGATCTA-3' and 5'-TAGATCTCCTAACCGA-3', with Bgl II sites. The ds DNA library was added as a competitor along with 10,000 CPM of a radiolabeled medium-strength CAP binding sequence, 5'-TTATGGAAGAGATATCACATTT-3', flanked by additional primer sequences, 5'-GTATGTCGAGATCTATCCAT-3' and 5'-TAATTTAGATCTCCTAACCG-3', to the left and the right, respectively. A library of random sequence oligos was obtained from Invitrogen and used as a template for primer extension with a 3' primer to make a double stranded library using TaqPro DNA polymerase (Cat No: CB-4050-7 from Denville Scientific Inc.). The resulting double stranded random library was added as a competitor to 50 nm CAP protein incubated with the radiolabeled medium strength CAP binding site. For the later rounds 25 nm CAP protein was used. The amount of library DNA was titrated until 50–80% of the radiolabeled complex was competed away. This was continued for 4 rounds.

For each round, after electrophoresis, the DNA-protein complexes were eluted using diffusion buffer from Qiagen (0.5 M ammonium acetate, 10 mM magnesium acetate, 1 mM EDTA, pH 8.0, 0.1% SDS) O/N at 37°C. The DNA was ethanol-precipitated and then PCR amplified. for 25 cycles. The cycling conditions were as follows. The initial denaturation was at 94°C for one minute. 25 cycles of amplification with 94°C for 30 secs, 40°C for one minute and 70°C for one minute followed the initial step. At the end, there was final primer extension at 74°C for 5 minutes and then a final hold at 4°C.

### Concatenation, cloning and sequencing

To obtain a large number of binding sequences, we concatenated the selected binding sequences to increase the sequencing throughput following [19]. The procedure for concatenation and cloning closely follows the SAGE procedure described in [43]. After the fourth round, the DNA was digested with BglII and gel purified using 4.5% regular agarose gels run in 1× TAE. The 36 mer band was cut out from gel and sliced further into very tiny pieces using a scalpel and extracted using quantum prep Freeze N Squeeze DNA Gel Extraction Spin columns (catalog 732–6166) from Bio-Rad laboratories. The purified 36-mers were then further spun through a microcon YM-10 from Millipore to eliminate further primer contamination and also to concentrate the sample. The concatenation procedure from [43] was followed. The concatemers (600–1200 base pair fragments) were gel purified and cloned into a BamH1 site of a pZero-1 vector (Invitrogen)[19] and transformed into DH10B *E. Coli *cells. The colonies were PCR amplified and were run on 1.5% gel to verify the fragment length. The colonies which contained insert sizes in the range of 600-1.2 kb were selected for sequencing. 5 ul of the PCR was treated with ExoSAP-IT, Cat.No 78201 from USB, incubated at 37°C for 15 minutes and inactivated by incubating at 85°C for 15 minutes. The reaction was cooled on ice and 3–4 ul of this was used for sequencing. The sequencing reaction was carried out using the M13 forward primer for 25 cycles (96°C for 10 secs, 50°C for 5 secs and 60°C for 4 minute and hold at 4°C). The reactions were cleaned using a CleanSEQ dye-terminator removal reagent from Agencourt Bioscience Corporation following the manufacturer's protocol. The samples were sent out for sequencing to Sequencing and Genotyping Core Facility, Genomics and Proteomics Core Laboratories, University of Pittsburgh.

### EMSA of various CAP binding sites predicted from QPMEME of known binding sites

Electrophoretic Mobility Shift Assays (EMSA) for CAP were carried out for several putative CAP binding sites in the *E. coli *genome as well as for one known CAP site and one generic site in *E. coli*. The putative CAP sites were chosen for an earlier unpublished study which aimed to estimate the false positive rate of QPMEME predictions based on known biologically functional binding sites. The sequences of the oligonucleotides are:

5'-**TAAAAAGTGTGACCCGGTTCACGTAGCGAT**-3' (TBS1),

5'-**GAATTCCTGCGCCTTTGCTCACAATCCAGA**-3' (TBS2),

5'-**TAAATATCGAGATAACGATCACAAAAACGA**-3' (TBS3),

5'-**GAAATTATGGAAGAGATATCACATTTCTAT**-3' (TBS4),

5'-**ATGCTAACGCGATTCCGCTCAAAAATCAGT**-3' (TBS5),

5'-**AGATCAATTTGATCTACATCTCTTTAACCA**-3'(TBS6),

5'-**CCTAATGAGTGAGCTAACTCACATTAATTG**-3' (Lac site),

5'-**GTCGCTGTTTTCCCGCCCGGTGTACGCCAC**-3' (Non-CAP site).

The oligonucleotides (both top and bottom strand) were obtained from Integrated DNA Technologies, INC. The top strand oligonucleotide (50 pmol) was 5'-end labeled using [*γ*-32P] ATP and T4 polynucleotide Kinase (New England Biolabs) according to the manufacturer's instructions. The labeled strand was purified from unincorporated [*γ*-32P] ATP using microspin G-50 column (Amersham Biosciences). A two-fold of unlabeled bottom strands were annealed to the 10 picomoles of top strand by heating the two at 95°C for 5 minutes and allowing them to cool gradually to room temperature overnight. The resulting double stranded radio-labelled DNA fragments (10,000 CPM) were incubated with various concentrations of CAP (0, 0.01, 0.1, 1, 10,100, 1000 nM) in a total volume of 20 ul containing 20 mM Tris-HCl, pH 8.0, 40 mM NaCl, 4 mM MgCl2, 0.1 mM EDTA, I mM DTT, 10 ug/ml sheared salmon sperm DNA, 0.2 mM cAMP and 6% glycerol. The complexes were fractionated using electrophoresis on a native 8% polyacrylamide (37.5:1) gel containing 2% glycerol, 0.1 mM cAMP in 1× TBE. The running buffer contained 2% glycerol and 20 uM cAMP in 1× TBE. The resulting gels were processed for analysis on a Molecular Dynamics Phosphoimager.

### Preprocessing of sequence data

96 concatemers were sequenced. For each of the concatemers, the subsequences consisting of between 24 and 32 nucleotides surrounded by restriction sites were extracted. Each occurrence of TAGATCTA was considered to be a restriction site, in addition to GGATCTA when it appeared before all of the other restriction sites and TAGATCC when it appeared after all of the other restriction sites. In all, 591 subsequences were extracted, along with their flanking restriction sites [see Additional file 5].

Each of the subsequences was then examined to determine whether CAP binding to the primer rather than the sequence from the random library contributed to the extraction of the sequence. The restriction sites were replaced by the original PCR primer sequences 5'-CTGTATGTCGAGATCTA-3' and 5'-TAGATCTCCTAACCGA-3', and the resulting sequences were scanned using an energy matrix constructed from 49 known CAP binding sites (and their reverse complements) taken from the DPInteract database [9]. The energy matrix was constructed using the QPMEME algorithm as described below. The matrix was used to assign estimated binding energies, or binding scores, to each subsequence of length 22. Sequences whose highest-scoring candidate binding site overlapped with the PCR primers were discarded, leading to a set of 94 sequences which contained a candidate CAP binding site within the subsequence from the random library. From the 94 sequences, 62 unique sites were extracted by selecting the site within the sequence, which was assigned the highest score by the energy matrix. Of these, 56 sites which had scores significantly beyond the threshold of -1 set by the algorithm were identified as candidate CAP binding sites while the remaining 6 sites, which were separated in energy from the rest of the sites by a significant gap, and which all scored below the threshold, were considered to have been selected due to non-specific binding and discarded.

The presence of sequences that are likely to have primers contributing to the binding of the TF, nearly 84% of the original dataset, seems unavoidable given that we perform selection at a high abundance of TF. We tried designing new primers that allow the least amount of binding, given our previous knowledge of CAP binding motif. We found that the condition of avoidance of a certain motif often makes the primer sequences self-similar leading to single stranded self-complementing loops. The combination of computational constraints like high free energy cost of partial overlap with the CAP binding motif, appropriate melting temperature, absence of self-looping and aperiodicity generated very few possibilities, and experiments using those computationally generated sequences had problems at stages past the SELEX steps. As a result, we decided to use the primers mentioned above, and use only a smaller subset of the data.

### SVM applied to SELEX data

We solve the dual [44] of the variance optimization problem mentioned above. We construct the matrix *M *in terms of the set of observed sequences *O *= {*S*^(1)^, *S*^(2)^, ..., *S*^(*N*)^} as follows:

Let us define the elements of the matrix *M *as,

$$M_{ab}={\hat{S}}^{\left(a\right)}•{\text{P}}^{-1}•{\hat{S}}^{\left(b\right)}\equiv \sum_{i=1}^{L}\sum_{\alpha =1}^{4}{\hat{S}}_{i\alpha }^{\left(a\right)}p_{\alpha }^{-1}{\hat{S}}_{i\alpha }^{\left(b\right)}$$

where ${\hat{S}}_{i\alpha }^{\left(a\right)}=S_{i\alpha }^{\left(a\right)}-P_{\alpha }$ and *P*_*αβ *_= *p*_*α *_*δ*_*αβ*_. We minimize $\frac{1}{2}\sum_{ab}\gamma_{a}M_{ab}\gamma_{b}-\sum_{a}\gamma_{a}$, subject to constraints *γ*_*a *_≥ 0 for each *a *= 1, ..., *N*.

The relation between the primal and the dual solution is given by $\varepsilon_{i\alpha }=\sum_{\alpha =1}^{N}\gamma_{a}{\text{P}}^{-1}•{\hat{S}}^{\left(b\right)}$. At the optimal point, for any *a*, such that, *γ*_*a *_> 0, we have $\varepsilon •{\hat{S}}^{\left(a\right)}$ = -1. If we think of sequences *S *as vectors in a vector space *V *and *H *= {*x *∈ *V*|*ε *• *x *= -1} a hyperplane separating the binding sequences from the non-binding ones, then *H *is "supported" by those observed sequences *S*^(*a*)^, which corresponds to non-trivial *γ*_*a*_.

For SELEX data *p*_*α *_is taken to be 0.25 for all *α *∈ {*A*, *C*, *G*, *T*}. In the case where biological binding sites are used as input to QPMEME, *p*_*α *_is set according to the frequency with which base *α *appears in the genomic background (in this case the non-ORF regions of the genome).

### Phylogenetic footprinting

Intergenic regions from *E. coli *were aligned to orthologous regions in the genome of the related bacterium *S. typhimurium*, in a manner similar to that described in [45]. An intergenic region in *S. typhimurium *was considered to be orthologous to a corresponding region in E. coli if the genes flanking the regions had the same names and relative orientation in both species. The regions were aligned using the ClustalW alignment program (reference) with the default parameters. This yielded 1,628 alignments from the full set of 3,475 intergenic regions in *E. coli*. 1,452 of the alignments contained an aligned sequence of length 22 or more, and were thus sufficiently long to contain a CAP binding site [see Additional file 6].

As described above, several methods were used to identify candidate CAP binding sites within the intergenic regions of the *E. coli *genome. For those candidate sites, which were located in a region with an *S. typhimurium *ortholog, the *S. typhimurium *sequence aligned to the *E. coli *site was extracted. In the cases when there were no gaps in the alignment, the extracted *S. typhimurium *sequence could be assigned an estimated binding energy using either a weight matrix or a QPMEME energy matrix.

## Authors' contributions

VHN performed all the experimental work. RAO contributed to the analysis of the binding sites that originated from the SELEX-SAGE data set. Both VHN and RAO helped in preparation of the manuscript. AMS designed and coordinated the experimental and bioinformatic aspects of the project and prepared the manuscript.

## Supplementary Material

Additional file 1 **Weight matrix extracted from DPInteract database sites.** The conventional weight matrix, obtained from the known CAP sites in the DPInteract database, is provided. The matrices are in tab-separated format with the order of the columns being A, T, G and C. Click here for file

Additional file 2 **Energy matrix extracted from DPInteract database sites.** The energy matrix, obtained by using QPMEME on known CAP sites in the DPInteract database, is provided. The matrices are in tab-separated format with the order of the columns being A, T, G and C. Click here for file

Additional file 3 **Weight matrix extracted from the SELEX dataset.** The conventional weight matrix, based on SELEX data presented in this paper, is provided. The matrices are in tab-separated format with the order of the columns being A, T, G and C. Click here for file

Additional file 4 **Energy matrix extracted from the SELEX dataset.** The energy matrix, obtained by training QPMEME on SELEX data presented in thin paper, is provided. The matrices are in tab-separated format with the order of the columns being A, T, G and C. Click here for file

Additional file 5 **SELEX sequences.** This file contains the 591 usable SELEX sequences (including flanking sequences) used in the study. Click here for file

Additional file 6 **Aligned intergenic sequences between *E. coli *and *S. typhimurium*.** The gzip compressed tar file includes the clustalw output and the sequences used in the study comparing CAP binding sites in *E. coli *and *S. typhimurium*. Click here for file
