# Supplementary material for: Better estimation of protein-DNA interaction parameters improve prediction of functional sites
Source: BMC Biotechnol. 2008 Dec 23;8:94. doi: 10.1186/1472-6750-8-94 (PMC2654563; doi:10.1186/1472-6750-8-94)
Supplement: Additional file 1 — Weight matrix extracted from DPInteract database sites. The conventional weight matrix, obtained from the known CAP sites in the DPInteract database, is provided. The matrices are in tab-separated format with the order of the columns being A, T, G and C. [file 1472-6750-8-94-S1.htm]

0.526491740510389 0.233756564495708 -0.630598059843926 -1.39631113936336
0.645953485715833 0.261703052773015 -1.07168803559026 -2.252597824319
0.341141386670699 0.0809511604758253 -0.145604797355571 -0.630598059843926
-1.49153553791487 0.950915258803318 -1.22088450285342 -0.393552482713135
-1.10130118515242 -0.539456941228888 1.23179755273703 -2.85310615102359
-1.87458082790734 0.991579531817919 -2.85310615102359 -0.145604797355571
-1.34388394030992 -0.906226969687586 1.33686030198694 -4.58496747867057
1.00477494660808 -1.49153553791487 -1.22088450285342 -0.723992999546512
-0.27169878067697 0.366277811272074 -0.0414253247233638 -0.202069131025611
-0.370373788296894 -0.181892003522745 -0.145604797355571 0.595377880878749
-0.370373788296894 0.233756564495708 -0.545185523106394 0.424581954774507
0.233756564495708 -0.370373788296894 0.424581954774507 -0.545185523106394
-0.181892003522745 -0.370373788296894 0.595377880878749 -0.145604797355571
0.366277811272074 -0.27169878067697 -0.202069131025611 -0.0414253247233638
-1.49153553791487 1.00477494660808 -0.723992999546512 -1.22088450285342
-0.906226969687586 -1.34388394030992 -4.58496747867057 1.33686030198694
0.991579531817919 -1.87458082790734 -0.145604797355571 -2.85310615102359
-0.539456941228888 -1.10130118515242 -2.85310615102359 1.23179755273703
0.950915258803318 -1.49153553791487 -0.393552482713135 -1.22088450285342
0.0809511604758253 0.341141386670699 -0.630598059843926 -0.145604797355571
0.261703052773015 0.645953485715833 -2.252597824319 -1.07168803559026
0.233756564495708 0.526491740510389 -1.39631113936336 -0.630598059843926
