# Supplementary material for: Better estimation of protein-DNA interaction parameters improve prediction of functional sites
Source: BMC Biotechnol. 2008 Dec 23;8:94. doi: 10.1186/1472-6750-8-94 (PMC2654563; doi:10.1186/1472-6750-8-94)
Supplement: Additional file 2 — Energy matrix extracted from DPInteract database sites. The energy matrix, obtained by using QPMEME on known CAP sites in the DPInteract database, is provided. The matrices are in tab-separated format with the order of the columns being A, T, G and C. [file 1472-6750-8-94-S2.htm]

-0.053790 -0.013607 0.009894 0.079446
-0.078657 -0.025083 0.052939 0.084576
-0.028388 -0.015681 0.029902 0.028515
0.059489 -0.121761 0.066884 0.015663
0.032487 0.004317 -0.136859 0.088071
0.059441 -0.083513 0.067096 -0.035187
0.068904 0.016621 -0.201442 0.088071
-0.130157 0.050442 0.062295 0.043375
0.007865 -0.017396 0.017518 -0.004884
0.016876 -0.025505 0.060520 -0.049081
0.007234 -0.018687 0.018383 -0.003200
-0.018687 0.007234 -0.003200 0.018383
-0.025505 0.016876 -0.049081 0.060520
-0.017396 0.007865 -0.004884 0.017518
0.050442 -0.130157 0.043375 0.062295
0.016621 0.068904 0.088071 -0.201442
-0.083513 0.059441 -0.035187 0.067096
0.004317 0.032487 0.088071 -0.136859
-0.121761 0.059489 0.015663 0.066884
-0.015681 -0.028388 0.028515 0.029902
-0.025083 -0.078657 0.084576 0.052939
-0.013607 -0.053790 0.079446 0.009894
