# Supplementary material for: Better estimation of protein-DNA interaction parameters improve prediction of functional sites
Source: BMC Biotechnol. 2008 Dec 23;8:94. doi: 10.1186/1472-6750-8-94 (PMC2654563; doi:10.1186/1472-6750-8-94)
Supplement: Additional file 3 — Weight matrix extracted from the SELEX dataset. The conventional weight matrix, based on SELEX data presented in this paper, is provided. The matrices are in tab-separated format with the order of the columns being A, T, G and C. [file 1472-6750-8-94-S3.htm]

0.604511107843314 -0.0645385211375712 -0.229862501562955 -0.748206957742973
0.715223132259145 0.337746934053213 -1.10758095865087 -1.35120304130862
0.544191317609791 -0.324049716622656 -0.0645385211375712 -0.428039430146703
-1.22199130982861 1.00181290531232 -3.10906095886099 -0.0271509890659509
-1.88528552723888 -1.49962304642689 1.29521628474771 -4.71849887129509
-1.22199130982861 1.24508047232335 -2.15354951383356 -1.88528552723888
-2.15354951383356 -2.52127429395888 1.34295804763292 -4.71849887129509
1.33359029762932 -3.10906095886099 -3.10906095886099 -1.88528552723888
-0.675447603460544 0.544191317609791 -0.607625007121783 0.230261019083074
-0.324049716622656 -1.00492680459079 -0.0271509890659509 0.679663830222658
-0.826678573184468 0.411399843627979 -0.374693449441411 0.337746934053213
0.411399843627979 -0.826678573184468 0.337746934053213 -0.374693449441411
-1.00492680459079 -0.324049716622656 0.679663830222658 -0.0271509890659509
0.544191317609791 -0.675447603460544 0.230261019083074 -0.607625007121783
-3.10906095886099 1.33359029762932 -1.88528552723888 -3.10906095886099
-2.52127429395888 -2.15354951383356 -4.71849887129509 1.34295804763292
1.24508047232335 -1.22199130982861 -1.88528552723888 -2.15354951383356
-1.49962304642689 -1.88528552723888 -4.71849887129509 1.29521628474771
1.00181290531232 -1.22199130982861 -0.0271509890659509 -3.10906095886099
-0.324049716622656 0.544191317609791 -0.428039430146703 -0.0645385211375712
0.337746934053213 0.715223132259145 -1.35120304130862 -1.10758095865087
-0.0645385211375712 0.604511107843314 -0.748206957742973 -0.229862501562955
