# Supplementary material for: Better estimation of protein-DNA interaction parameters improve prediction of functional sites
Source: BMC Biotechnol. 2008 Dec 23;8:94. doi: 10.1186/1472-6750-8-94 (PMC2654563; doi:10.1186/1472-6750-8-94)
Supplement: Additional file 4 — Energy matrix extracted from the SELEX dataset. The energy matrix, obtained by training QPMEME on SELEX data presented in thin paper, is provided. The matrices are in tab-separated format with the order of the columns being A, T, G and C. [file 1472-6750-8-94-S4.htm]

-0.011756 -0.008268 -0.001654 0.028198
-0.022527 -0.028141 0.040405 0.026760
-0.035500 0.008149 0.013990 0.022267
0.037619 -0.066993 0.050577 -0.011639
0.030650 0.038183 -0.141821 0.050577
0.050577 -0.093253 0.028285 0.028285
0.031665 0.040010 -0.145588 0.050577
-0.106836 0.050577 0.050577 0.023998
0.022042 -0.022481 0.008737 -0.008155
-0.005412 0.031690 0.012542 -0.047375
0.035600 -0.029350 0.005804 -0.014088
-0.029350 0.035600 -0.014088 0.005804
0.031690 -0.005412 -0.047375 0.012542
-0.022481 0.022042 -0.008155 0.008737
0.050577 -0.106836 0.023998 0.050577
0.040010 0.031665 0.050577 -0.145588
-0.093253 0.050577 0.028285 0.028285
0.038183 0.030650 0.050577 -0.141821
-0.066993 0.037619 -0.011639 0.050577
0.008149 -0.035500 0.022267 0.013990
-0.028141 -0.022527 0.026760 0.040405
-0.008268 -0.011756 0.028198 -0.001654
